# Study of clinical, haematological and cytogenetic profile of patients with acute erythroid leukaemia

**DOI:** 10.3332/ecancer.2017.712

**Published:** 2017-01-10

**Authors:** Jacob Abraham Linu, MS Namratha Udupa, DS Madhumathi, KC Lakshmaiah, K Govind Babu, D Lokanatha, MC Suresh Babu, KN Lokesh, LK Rajeev, AH Rudresha

**Affiliations:** Kidwai Memorial Institute of Oncology, Bengaluru 560029, India

**Keywords:** clinicohematological profile, cytogenetics, acute erythroid leukemia

## Abstract

**Background:**

Acute erythroid leukaemia (AEL) is a rare subtype of acute myeloid leukaemia (AML), constituting <5% of all the cases of AML. The World Health Organization (WHO) in 2001 classified AEL into two types: (1) erythroid/myeloid leukaemia which required ≥50% erythroid precursors with ≥20% of the non-erythroid cells to be myeloid blasts and (2) pure erythroleukemia (pEL) with ≥80% erythroblasts. The WHO 2008 classification kept these subcategories, but made erythroleukemia a diagnosis of exclusion. There are very few studies on the clinico haematological and cytogenetic profile of this disease, considering the rarity of its occurrence and poor prognosis.

**Materials and methods:**

This study was done by retrospective analysis of data from 32 case files of patients diagnosed with AEL. Clinical details noted down were the demographic profile, peripheral blood smear details and bone marrow examination details: (1) blasts-erythroblasts and myeloblasts, (2) dysplasia in the cell lineages and (3) cytogenetic abnormalities.

**Results:**

The most common presenting symptom was fever. Pancytopenia at presentation was seen in 81.25% of patients. Dysplasia was observed in bone marrow in 100% of erythroblasts and in 40% of myeloblasts in erythroid/myeloid subtype. In pure myeloid subtype, myeloid and megakaryocytic dysplasias were not obvious. Complex karyotype was noticed only in patients of pEL.

**Conclusion:**

AEL is a rare group of heterogeneous diseases with many neoplastic and non-neoplastic conditions mimicking the diagnosis. The clinical presentation and cytogenetics are also non-specific, presenting additional challenges to the diagnosis.

## 1 Background

Acute erythroid leukaemia (AEL) is a rare subtype of acute myeloid leukaemia (AML), constituting <5% of all the cases of AML [1]. The definition of this disease has been revised multiple times. The probability of existence of this disease was first deduced by Coppelli in 1912. He documented the first case of AEL [2]. In 1928, Di Guglielmo described the first case of pure erythroid leukaemia and documented it as eritremia acuta, which was later designated as Di Guglielmo disease [3]. Probability of pathologic evolution of acute erythroid myeloid leukaemia was first hypothesised by Damshek as development from phase of non-malignant erythremic proliferation through phase of erythroleukemia to myeloblastic leukaemia, and he named it Di Guglielmo syndrome [4].

AEL was designated as AML-M6 by the FAB Cooperative Group in 1976 and diagnosis required presence of erythroid precursors ≥30% and dyserythropoiesis ≥10%. Following the amendments brought to the FAB classification in 1985, AML-M6 was to be defined by the criteria: ≥50% erythroblast of all nucleated cells, prominent dyserythropoiesis and ≥30% of myeloblast of the rest of non-erythroid precursors. In this, paradoxically ignored was the rare subtype of pure erythroid leukaemia which was classified under refractory anaemia with excess blasts in transformation [5]. The World Health Organization (WHO) in 2001 classified AEL into two types: (1) erythroid/myeloid leukaemia which required ≥50% erythroid precursors with ≥20% of the non-erythroid cells to be myeloid blasts and (2) pure erythroleukemia (pEL) with ≥80% erythroblasts. WHO 2008 classification kept these subcategories, but made erythroleukemia a diagnosis of exclusion [2]. Despite these amendments to the definition, there is considerable overlap with the diagnosis of AEL with various myelodysplastic syndromes (MDSs) [6]. The latest amendment to the WHO classification in the year 2016 was a big stroke to the all the confusions surrounding this entity. The entity of AEL is being proposed to be removed [7–9]. The myeloblasts need to be counted as a percentage of the total marrow cells. This will be mostly <20% and will qualify to be called as MDS, mostly the subtype with excess blasts. This study was made keeping in line with the 2008 classification as the 2016 classification was too recent at the time of conducting this study.

There are very few studies on the clinico haematological and cytogenetic profile of this disease, considering the rarity of its occurrence and poor prognosis.

## 2 Materials and methods

This study was done by retrospective analysis of data from case files of patients diagnosed with AEL at the Kidwai Memorial Institute of Oncology over a period of 19 years from January 1997 to March 2016. A total of 1223 of AML were diagnosed in this period of which 32 patients had AEL (2.6%).

Clinical details noted down were the demographic profile: (1) age, (2) gender, (3) presenting symptoms and signs with duration, (4) past history of MDS/MPN or exposure to chemotherapy/radiotherapy and (5) family history. Also noted down were the peripheral blood smear details and bone marrow examination details: (1) blasts-erythroblasts and myeloblasts, (2) dysplasia in the cell lineages and (3) cytogenetic abnormalities. Peripheral smear and bone marrow aspiration slides of all the patients were reviewed, and diagnosis was revisited in view of new amendments to WHO definition of the entity. Diagnosis of AEL was made according to the WHO 2008 definition. Cytogenetic analysis was performed by conventional karyotyping of at least 10 metaphases, and the nomenclature system adopted was the International System for Chromosome Nomenclature (1985 and 1995).

## 3 Results

### 3.1 Clinical profile

32 AEL patients were diagnosed in this period in 19 years of which 28 were of myeloid/erythroid subtype (erythroleukemia-M6a) and 4 were of pure erythroid subtype (pEL-M6b). Of these patients, 22(68.75%) were males and 10(31.25%) were females. In the group of patients with pEL, three were males and only one was a female patient. Median age was 36.3 years (Table 1).

The most common presenting symptom was fever seen in 22(68.75%) patients, whereas the most common symptom present was easy fatiguability seen in 28(87.5%) patients. Other prominent presenting symptoms were mild mucocutaneous bleeding seen in 5(15.6%) patients and lymphadenopathy seen in 4(12.5%) patients. Signs on examination were pallor in 25(78.12%) patients, jaundice in 10(31.25%) patients and organomegaly in 10(31.25%) patients: hepatosplenomegaly in 2(6.25%), only hepatomegaly in 5(15.62%) and only splenomegaly in 3(9.3%) patients. Most patients had an ECOG PS of 2. At presentation, most of them required blood/component transfusions. Mean total bilirubin was 2.7 g/dl (0.8–4.7) (Table 2).

### 3.2 Haematological profile

#### 3.2.1 Peripheral smear

Median haemoglobin was 7.8 g/dl (4–11.2), median total leucocyte count was 4400/mm^3^ (1800–19200) and median platelet count was 92000/mm^3^ (10000–1.8 lacs). Pancytopenia at presentation was seen in 81.25% of patients.

#### 3.2.2 Bone marrow morphology

**Figure 1.**
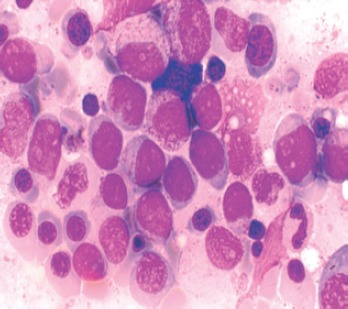
Hyperplastic marrow showing erythroblasts at all stages of maturation and are more than 50% of marrow cells. Myeloblasts are also present.

**Figure 2.**
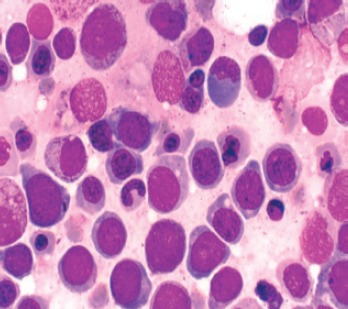
Hyperplastic marrow showing dysplasia of erythroblasts-macronormoblastic reaction, bi/multinucleated forms and karryorhexis.

**Figure 3.**
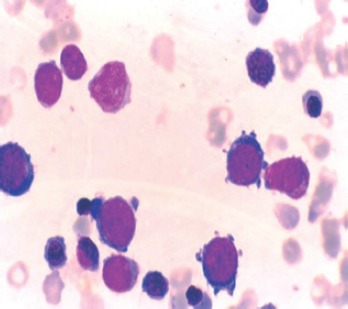
Erythroblasts constituting more than 80% of cells in the marrow-pure erythroid leukemia.

**Figure 4.**
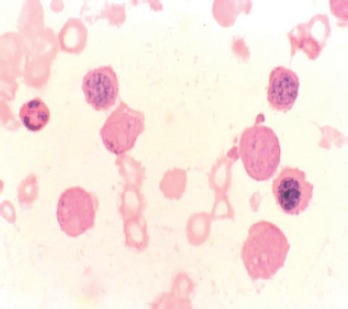
Erythroblasts demonstrating coarse granules on PAS staining.

Bone marrow aspirate morphological examination had a mean erythroblast count of 65%(55–95), prominent shift to left with various dysplastic features: megaloblastic forms (22 patients–68.75%), nuclear budding (19 patients–59.37%), bi and multinucleated forms (21 patients–65.6%), cytoplasmic vacuolization (17 patients–53.1%), nuclear bridging (12 patients–37.5%) and karyorrhexis (7 patients–21.8) (Table 3). Erythroid precursor dysplasia was observed in 100% of cases, whereas granulocytic and megakaryocytic dysplasias were seen in 40% and 30% of patients, respectively, in erythroid/myeloid subtype. In pure erythroid subtype, granulocytic and megakaryocytic dysplasias were not obvious (Figures 1–4).

#### 3.2.3 Cytogenetic profile

Results of cytogenetics by conventional karyotyping is as detailed below. Complex karyotype was noticed only in patients of pEL (Table 4).

## 4 Discussion

AEL is a hematopoietic neoplasm affecting the erythroid and myeloid precursor cells of bone marrow. The definition of AEL has undergone metamorphoses in accordance with the expanding knowledge of various other disease entities which are pathological mimics of this disease and the biologic and clinical behaviour of the disease. The diagnosis of AEL is one of exclusion. The entities to be excluded are AML-myelodysplasia-related changes (AML-MRC), therapy-related AML (t-AML), AML with erythroblast proliferation and recurrent genetic abnormalities, erythroblast phase of myeloproliferative neoplasms (MPNs) and reactive erythroid hyperplasia after erythropoietin (EPO) treatment [2].

Diagnosis of AML-MRC requires the presence of at least 20% of blasts in the blood/marrow with one of the following: history of MDS/MPN, MDS-related cytogenetic abnormalities and absence of any of recurrent cytogenetic abnormalities, or presence of a minimum of 50% of dysplastic cells in at least two lineages with absence of history of exposure to cytotoxic agents: (1) alkylating agents and (2) topoisomerase II inhibitors [10]. t-AML has a history of exposure to alkylating agents/topoisomerase II inhibitors, specific cytogenetic abnormalities and a median time to development of 3–5 years [11]. Some MPNs are associated with erythroblastic phase characterised by marked erythroid hyperplasia of the bone marrow with nucleated RBCs in the peripheral blood [12]. There are other non-neoplastic differential diagnoses which need to be ruled in certain situations. These are EPO treatment, vitamin B12 or folate deficiency, exposure to toxins like benzene and parvovirus infection [2, 13]. EPO treatment can mimic pure erythroid leukaemia especially. One of the major differentiating features of the two, apart from history of treatment with EPO, would be that pure erythroid leukaemia presents with severe anaemia and circulating blasts whereas EPO-treated patients will have their anaemia corrected.

Incidence of AEL in our population of AML patients was 2.6%. This is less than the incidence (4.3%) quoted in Attili *et al* from the same institute [14]. In this, the share of pEL was 12.5%. There was not even a single case of secondary AML. Various studies [15–18] have quoted similar incidence. Median age in our patients was 36.3 years, which is far lesser than age (56–66 years) in various other studies [15, 16]. Some studies have also quoted bimodal distribution of age [6, 19, 20]. The first small peak is found to be below the age of 20 years. We hypothesised this difference from other literature for two reasons. First, the median age incidence of all unclassified types of AML from an unpublished data from this institute is 37 years, which is quite an alarming development. There is also evidence for the earlier occurrence of other cancers like breast cancer in India [21]. Whether we can attribute this probable age shift to rapid urbanisation is an open-ended question which calls for further studies. Second, there was no case of secondary AEL which has an incidence of 20%–30% otherwise among secondary AMLs [14]. The disease has a male preponderance akin to the results from the other studies [15, 16, 19].

Most common presenting symptom was fever which was similar to that in other studies [18, 22]. AEL can present with non-specific clinical features [6, 14]. Symptoms could be due to anaemia and resultant asthenia [20]. Mean haemoglobin in our study was 7.8 g/dl which was consistent with other studies [6, 18]. Up to 30% of patients can have bleeding symptoms or organomegaly at presentation [6, 23]. However, some studies have incidence of organomegaly up to 70% [17]. In this series, organomegaly was seen in 15.6%. Lymphadenopathy was seen in 12.5% of patients which is different from the absence of it in other studies [17–19]. One surprising feature was the occurrence of jaundice in about 31.25% of all AEL patients, but in all of the four patients of pEL. This is rarely though reported in other studies [24, 25]. Pancytopenia was seen in 81.25% of patients in this study which was higher than that in other studies. Up to 50% of patients have no blasts on peripheral smear [20]. Bleeding manifestations are reported in about one-third of patients [20, 26]. In this series, only minor mucocutaneous bleeding was noticed in 15% of patients.

Bone marrow was hypercellular with prominent dysplasia in erythroid lineage [20, 27]. In the presence of prominent dysplasia, erythroid prominent MDS (with >50% erythroblasts) and AML-MRC are two closely related entities. Patients of MDS, in general, have anaemia and dysplasia which depicts a stress on the bone marrow. Diagnosis of MDS requires a blast percentage of <20% of all nucleated cells and also <20% of non-erythroid precursors [6]. The latter is to alleviate the risk of over-diagnosis of low blast count subset of AEL, which behaves more like MDS than AEL. This is a small subset of patients with high erythroid precursors (70%–90%) which allows the myeloblasts to be >20% of the non-erythroid precursors [1, 6]. In addition, it has been shown that enumerating blasts as a percentage of non-erythroid cells are superior as compared with the percentage of all nucleated cells in risk stratification of the patients [28, 29]. At least one cell lineage dysplasia was seen in all the cases, most common being in erythroid lineage followed by megakaryocyte and myeloid lineages. Single lineage dysplasia was observed in 12(37.5%), double lineage in 15(46.8%) and triple lineage in 5(15.6%) of patients. In pure erythroid subtype, dysplasia in non-erythroid lineages was a minor finding. Only dysplasia in erythroid lineage was seen in eight patients (25%), which is higher than that (10%) observed in the study by Domingo-Claros *et al* [30]. Changes were non-specific, and megaloblastic forms were most commonly seen.

No cytogenetic abnormality is specific for AEL. Four patients with cytogenetic abnormalities involving deletions in chromosomes 5 and 7, who were earlier classified as AEL from a study at the same institute [14], were reclassified as AML-MRC in the wake of new and more stringent definition of WHO 2008. However, all four patients of pure erythroid subtype harboured complex karyotype. This is similar to other articles on pure erythroid leukaemia [31, 32]. Complex karyotype was defined as having at least three cytogenetic abnormalities, but only in the absence of WHO termed recurrent inversions or translocations [33]. This could probably be explained by the younger patient population in this study, and it is known that the incidence of complex karyotype increases with age at diagnosis [34].

Of the 32 patients studied, 21(65%) received only the best supportive care; 4(12.5%) received low dose subcutaneous cytarabine; 3(9.3%) received decitabine; 4 received intensive 3 + 7 induction chemotherapy of infusional daunorubicin and cytarabine. The overall survivals in the respective subgroups were 2, 4, 5 and 14 months. The median follow-up period was 7 months.

## 5 Conclusion

AEL is a rare group of heterogeneous diseases with many neoplastic and non-neoplastic conditions mimicking the diagnosis. The clinical presentation and cytogenetics are also non-specific offering additional challenges to the diagnosis. Though it has an inherent biological relationship with MDSs, it has a worse clinical behaviour. Amidst the thoughts of phasing out this diagnosis and merging it with MDS, one must appreciate its differences in clinical outcomes. A probable age shift with regard to a younger population calls for more studies in this regard.

## Conflict of interest

None.

## Figures and Tables

**Table 1.** Clinical Profile, Peripheral Smear and Bone Marrow Morphology.

Clinical Characteristics	AEL	pEL
Median age	36.3 years (27–51)	42 years (35–67)
Gender distribution	22 males: 10 females: (2.2:1–M:F)	3 males: 1 female:: 3:1
History of previous chemo/radiotherapy	None	None
Family history	None	None
Median leucocyte count	5100/mm^3^ (3300–19200)	2900/mm^3^ (1800–12000)
Median neutrophil count	2200 (2000–8000)	1500 (1100–5000)
Median platelet count	95000/mm^3^ (30000–1.8 lacs)	52000/mm^3^ (10000–70000)
Median haemoglobin	8.2 g/dl (5.5–11.2)	6.5 g/dl (4–9.2)
Median bone marrow erythroblast percentage	65	88
Median bone marrow myeloblast percentage (from total nucleated cells)	15	10
Median bone marrow myeloblast percentage (from non-erythroid cells)	25	20
Percentage with erythroid dysplasia	100	100
Percentage with granulocytic dysplasia	40	10
Percentage with megakaryocytic dysplasia	30	0

**Table 2.** Symptoms and Physical Findings.

Presenting Symptom/Signs	Prevalence (%)
Fatigue	87.5
Pallor	78.12
Fever	68.75
Generalised myalgia	40
Jaundice	31.25
Mucocutaneous bleeding	15.6
Hepatomegaly	15.6
Lymphadenopathy	12.5
Splenomegaly	9.3
Hepatosplenomegaly	6.25

**Table 3.** Dysplasia in Bone Marrow Morphology.

Dysplastic Features	Prevalence (%)
Erythroid dysplasia-Megaloblastic forms	68.75
Erythroid dysplasia-Bi and multinucleated forms	65.6
Erythroid dysplasia-Nuclear budding	59.37
Erythroid dysplasia-Cytoplasmic vacuolization	53.1
Erythroid dysplasia-Nuclear bridging	37.5
Erythroid dysplasia-Karyorrhexis	21.8
Granulocytic dysplasia-hypolobated, (this is not a dysplastic trait), hypo and degranulated neutrophils	40
Megakaryocytic dysplasia-small in size, hypo and monolobulated	30

**Table 4.** Cytogenetic Profile.

Cytogenetics	Prevalence
Normal karyotype 10 patients: 46,XY 5 patients: 46,XX	15(46.8)
2 patients: 46,XY(–20) 1 patient: 46,XX(–20)	3(9.3)
3 patients: 46,XY/t (3, 5)	3(9.3)
1 patient: 47,XX/Trisomy 13	1(3.12)
1 patient: 47,XY/Trisomy 8	1(3.12)
Complex karyotype 46,XY(–3, –5, –11,13) 46,XY(–3, +13, –20) 46,XY(–9, –11, –20) / t(3, 5) 46,XX(–15, –11, –20)	4(12.5)
No information available (Failed cytogenetics)	5(15.4)
